# Xinli formula’s efficacy in heart failure: analysis via Guangdong TCM hospital database

**DOI:** 10.3389/fphar.2025.1661625

**Published:** 2025-11-17

**Authors:** Haiming Cao, Jiongming Zhang, Tong Zhang, Xiankun Chen, Tong Liu, Wenjing Xu, Yi Zhu, Wei Jiang, Weihui Lu

**Affiliations:** 1 Second Clinical Medical College, Guangzhou University of Chinese Medicine, Guangzhou, Guangdong, China; 2 Department of Cardiology, The Second Affiliated Hospital of Guangzhou University of Chinese Medicine, Guangzhou, Guangdong, China; 3 Huizhou Hospital of Chinese Medicine, Huizhou, Guangdong, China; 4 Clinical Research Center, The Second Affiliated Hospital of Guangzhou University of Chinese Medicine, Guangzhou, Guangdong, China; 5 Academician Chen Keji Workstation, The Second Affiliated Hospital of Guangzhou University of Chinese Medicine, Guangzhou, Guangdong, China; 6 Chinese Medicine Guangdong Laboratory, Hengqin, Guangdong, China; 7 State Key Laboratory of Traditional Chinese Medicine Syndrome, Guangdong Provincial Hospital of Chinese Medicine, Guangzhou University of Chinese Medicine, Guangzhou, Guangdong, China; 8 Guangdong Provincial Key Laboratory of Chinese Medicine for Prevention and Treatment of Refractory Chronic Diseases, Guangzhou, China

**Keywords:** heart failure, traditional Chinese medicine, Xinli formula, efficacy, hospital database

## Abstract

**Objective:**

To evaluate the efficacy of the “Xinli Formula” in improving cardiac function, symptoms, and quality of life in patients with heart failure using a hospital database.

**Methods:**

A retrospective study included 210 patients (2017–2022) divided into Xinli Formula group (n = 107) and non-Xinli Formula group (n = 103). Outcomes included cardiac function parameters, N-terminal pro-B-type natriuretic peptide (NT-proBNP) levels, 6-min walk distance (6MWD), quality of life (Minnesota Living with Heart Failure Questionnaire, MLHFQ), and inflammatory markers. Data were analyzed using SPSS 26.0.

**Results:**

The Xinli Formula group showed significant improvements in Left Ventricular Ejection Fractions (LVEF) (38% → 42%), Left Ventricular End-Systolic Diameter (LVESD) (45 mm → 40 mm), Left Ventricular End-Diastolic Diameter (LVEDD) (60 mm → 55 mm), and 6MWD (350 m → 400 m). MLHFQ scores decreased from 45 to 30. Inflammatory markers and NT-proBNP levels also improved significantly.

**Conclusion:**

The Xinli Formula significantly improves cardiac function, exercise tolerance, and quality of life in patients with heart failure and may be an effective adjunctive therapy. Further studies are needed.

## 1 Introduction

Heart failure is a complex clinical syndrome primarily caused by abnormalities in cardiac structure or function, or both, leading to impaired ventricular contraction and/or relaxation (Arrigo et al., 2020). The etiology of heart failure is diverse, including coronary heart disease, hypertension, cardiomyopathy, and other conditions that can lead to changes in cardiac structure and function, thereby triggering heart failure (Gatzov et al., 2021). The clinical manifestations of heart failure mainly include dyspnea, fatigue, edema, and other symptoms that severely affect the quality of life and survival rate of patients (Bozkurt et al., 2021). Although guideline-directed drugs and devices reduce mortality, many patients remain symptomatic, are re-hospitalised, or experience quality-of-life impairment, underscoring the need for safe adjunctive therapies.

In recent years, numerous clinical studies have shown that traditional Chinese medicine (TCM) has unique advantages in the treatment of heart failure (Tang and Huang, 2013; Shao-Mei et al., 2022). TCM’s syndrome differentiation and treatment of heart failure can not only effectively alleviate patients’ clinical symptoms but also significantly improve cardiac function and show good effects in improving prognosis (Wang et al., 2022). TCM, through its holistic concept and syndrome differentiation, targets the different causes, pathogenesis, and syndromes of heart failure, adopting personalized treatment plans to achieve both symptomatic and radical treatment.

Academician Chen Keji, drawing on years of clinical experience, has summarized the “Xinli Formula,” an empirical prescription for treating heart failure (Liu et al., 2023). This formula, composed of Curcuma zedoaria, Astragalus membranaceus, Plantago asiatica, Panax ginseng, Astragalus membranaceus (prepared), and Cornus officinalis. Astragali Radix (Astragalus membranaceus Bunge): Contains astragalosides and polysaccharides that exert positive inotropic, antioxidant, and anti-inflammatory effects. Ginseng Radix (Panax ginseng C.A.Mey.): Rich in ginsenosides; enhances myocardial energy metabolism and prevents cardiomyocyte apoptosis. Curcumae Rhizoma (Curcuma zedoaria Roscoe): Provides curzerenone with anti-inflammatory and anti-fibrotic actions; improves micro-circulation. Corni Fructus (Cornus officinalis Siebold & Zucc.): Contains loganin with potent antioxidant activity; protects vascular endothelium. Plantaginis Semen (Plantago asiatica L.): Contains plantago-polysaccharides that induce diuresis and confer renal protection. In clinical practice, the “Xinli Formula” has shown good efficacy, but its effectiveness and safety still need further validation in large-scale clinical studies.

Traditional clinical research faces many problems in case data collection, such as low efficiency, high error rates in manual data collection, and difficulty in ensuring data integrity. These issues seriously affect the scientific nature and reliability of research. With the development of information technology, clinical research based on databases has gradually become a new trend (Wu et al., 2021). The Heart Failure Specialized Disease Database Platform of Guangdong Provincial Hospital of Chinese Medicine has provided high-quality, standardized, and structured data support for this study, effectively solving the limitations of traditional data collection.

Therefore, this study, based on the Heart Failure Specialized Disease Database Platform of Guangdong Provincial Hospital of Chinese Medicine, adopts a retrospective research method to explore the efficacy of the “Xinli Formula” on patients with heart failure. By analyzing the clinical data in the database, this study assesses the effects of the “Xinli Formula” in improving cardiac function, alleviating symptoms, enhancing quality of life, and improving prognosis, providing scientific evidence for the treatment of heart failure with traditional Chinese medicine and further promoting the application and development of TCM in the treatment of heart failure.

## 2 Objects and methods

### 2.1 Study participants

**FIGURE 1:**
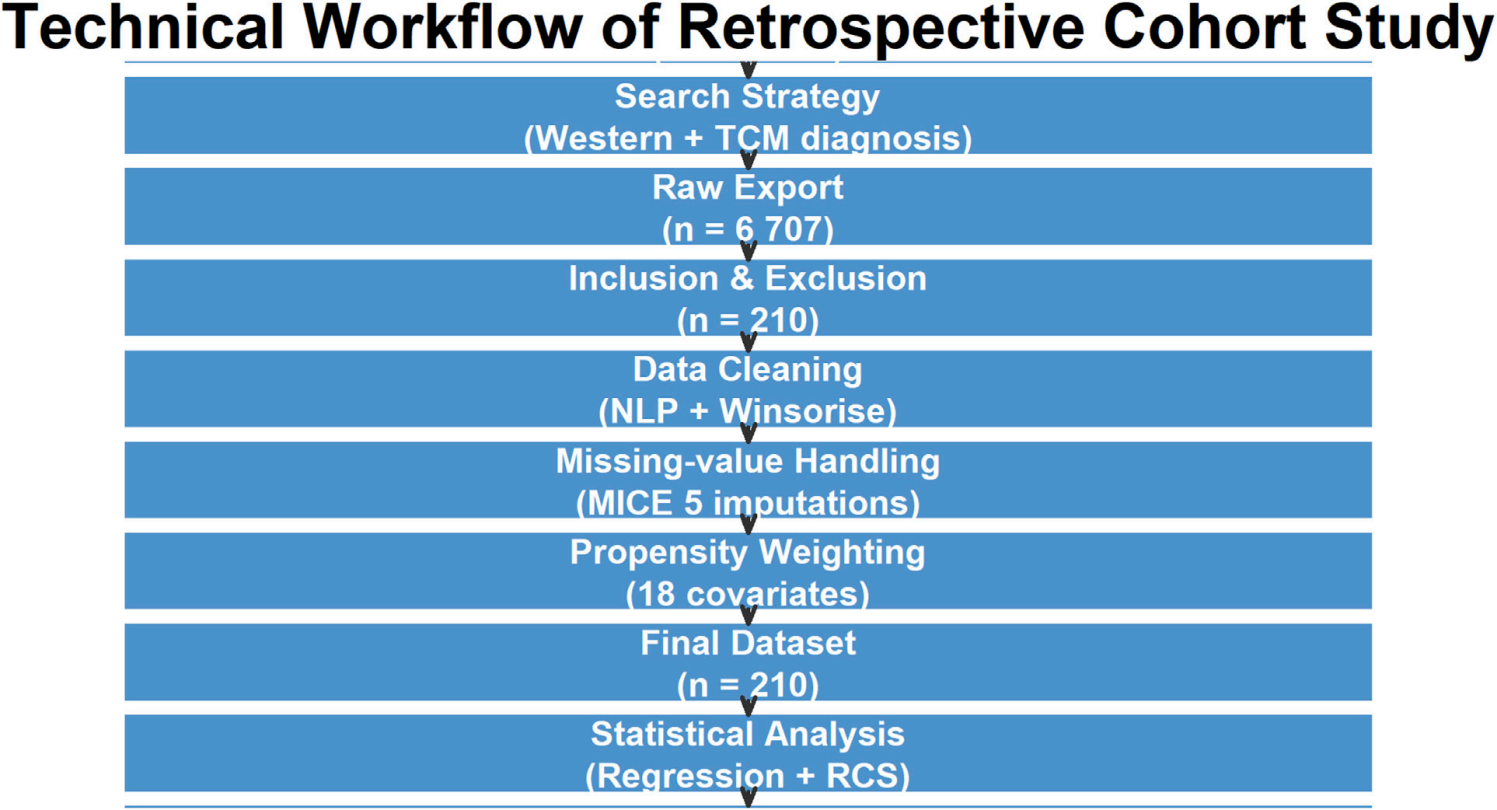
Technical workflow of data extraction, cleaning, and analysis. HF = heart failure; NLP = natural-language processing; MICE = multiple-imputation chained equations; RCS = restricted cubic splines.

This study employed a retrospective cohort design. Cases were sourced from the Heart Failure Specialized Disease Database Platform of Guangdong Provincial Hospital of Chinese Medicine (CM). We included patients admitted to the Heart Failure Center and ICU of the Guangdong Provincial Hospital of Chinese Medicine from January 2017 to December 2022. A total of 210 patients were included in the study, with 107 in the Xinli Formula group and 103 in the non-Xinli Formula group, based on the inclusion and exclusion criteria (Figure 1).

### 2.2 Inclusion criteria

- Patients admitted to the Heart Failure Center and ICU of the Ersha Island Branch of Guangdong Provincial Hospital of Chinese Medicine from January 2017 to December 2022.
- Diagnosis of heart failure was made in accordance with the 2021 ESC Guidelines for the diagnosis and treatment of acute and chronic heart failure (McDonagh et al., 2021).
- Typical clinical manifestations (e.g., dyspnea, fatigue, edema).
- Evidence of cardiac structural or functional abnormalities, such as reduced Left Ventricular Ejection Fraction (LVEF ≤40%) or increased Left Ventricular End-Diastolic Diameter (LVEDD) (Chai et al., 2023).
- Elevated levels of cardiac biomarkers (e.g., B-type natriuretic peptide (BNP) or N-terminal pro-B-type natriuretic peptide (NT-proBNP) (Lowe, 2006).

### 2.3 Exclusion criteria

- Patients with severe hepatic or renal insufficiency, terminal cancer, or other conditions severely affecting quality of life.
- Patients with severe allergic reactions to the drugs used in this study.
- Patients with serum creatinine levels greater than 160 umol/L at admission.
- Patients who used both Xinli Formula and other traditional Chinese medicine prescriptions that did not contain Xinli Formula during hospitalization.

### 2.4 Study methods

#### 2.4.1 Determination of the Heart Failure Specialized Disease Database Platform search formula

Search Fields: Western medicine discharge diagnosis (OR linked): Heart failure (including “chronic heart failure,” “acute heart failure”), heart failure (including “left heart failure,” “right heart failure,” “biventricular failure”), cardiac insufficiency (including “left ventricular dysfunction,” “right ventricular dysfunction,” “biventricular dysfunction,” “chronic left ventricular dysfunction”), ventricular dysfunction, atrial dysfunction, cardiac function (including “Class IV cardiac function,” “Class III cardiac function,” “Class II cardiac function,” “Class I cardiac function”), ventricular failure (including “left ventricular failure,” “right ventricular failure”), atrial failure (including “left atrial failure,” “right atrial failure”).

Search Fields: Traditional Chinese medicine discharge diagnosis (OR linked): Heart failure, heart water disease.

Search Fields: Western medicine diagnostic codes (OR linked).

Search Formula: ① OR ② OR ③.

#### 2.4.2 Establishment of the heart failure clinical data database

Using the specialized disease database platform of Guangdong Provincial Hospital of Chinese Medicine, initial Excel data were exported based on the above search formula and de-identified. After screening according to the inclusion and exclusion criteria, an Excel database was established. This database included information on patients’ gender, age, length of hospital stay, comorbidities, concomitant medications, clinical symptoms, signs, laboratory examination indicators, follow-up time points, and follow-up methods. All variables were extracted from the electronic health records (EHR) of Guangdong Provincial Hospital of Chinese Medicine by two independent clinicians using a pre-defined case-report form. The following fields were downloaded from the hospital information system (HIS) and heart-failure module: demographics, admission/discharge dates, medical history, medication orders, laboratory results (NT-proBNP, creatinine, electrolytes, liver function, blood count), echocardiographic reports, 6-min walk distance, and Minnesota Living with Heart Failure Questionnaire (MLHFQ) scores. Data completeness was checked manually; records missing any of the primary end-point values baseline or day-7 NT-proBNP, LVEF, 6-min walk distance (6MWD) were excluded. As this is a retrospective registry study, no *a priori* sample-size calculation was performed. Post-hoc power was estimated from the observed 7-day treatment-success rates (64.5% vs. 45.6%). Using the standard formula for two independent proportions with two-sided α = 0.05 and β = 0.20, 91 participants per arm (total 182) are required. The present dataset (n = 210; 107 vs. 103) yields 83% power, sufficient to detect the 19% absolute difference detected in this cohort. Power for continuous secondary end-points (6MWD and LVEF) exceeded 80%.

#### 2.4.3 Missing-value strategy

NT-proBNP, 6MWD, echocardiographic parameters: if more than one value was available we used the measurement closest to discharge; if none, the case was excluded.

Laboratory tests (haemoglobin, creatinine, electrolytes): single missing value was imputed with the median of the same sex/age stratum (<2% of all tests).

MLHFQ: one missing post-treatment questionnaire (Xinli group) was handled by last-observation-carried-forward (baseline score carried to day 7); sensitivity analysis excluding this patient did not change the conclusions.

### 2.5 Study design

This study adopted a retrospective cohort design, with the study group being patients treated with “Xinli Formula” and the control group being those not treated with “Xinli Formula.”

#### 2.5.1 Intervention measures

Study Group: Standard treatment guided by guidelines + “Xinli Formula,” which includes Plantago asiatica 30 g, Curcuma zedoaria 20 g, Panax ginseng 10 g, Astragalus membranaceus 30 g, Astragalus membranaceus (prepared) 30 g, and Cornus officinalis 20 g. The treatment duration was 1 week. No changes in composition or dosage were allowed, ensuring full consistency of the intervention. The hospital pharmacy produced a standardized 20 g granule dissolved in warm water, twice daily for 1 week.

Herb quality: Astragali Radix: Dried root of Astragalus membranaceus (Fisch.) Bunge var. mongholicus (Bunge) P.K.Hsiao; astragaloside IV ≥0.040%. Ginseng Radix et Rhizoma: Dried root/rhizome of Panax ginseng C.A.Mey.; total ginsenosides Rg1+Re ≥0.30%, ginsenoside Rb1 ≥0.20%. Curcumae Rhizoma: Dried rhizome of Curcuma phaeocaulis Val., C. kwangsiensis S.G.Lee & C.F.Liang, or C. wenyujin Y.H.Chen & C. Ling; essential oil ≥1.5%. Corni Fructus: Dried ripe sarcocarp of Cornus officinalis Siebold & Zucc.; loganin ≥0.60%. Plantaginis Semen: Ripe seed of Plantago asiatica L. or *P. depressa* Willd.; plantenolic acid ≥0.40%.

Preparation: Plantaginis Semen is wrapped in gauze, soaked together with the other herbs for 30 min. First decoction: eight volumes of water, gentle boil 30 min after boiling. Second decoction: six volumes of water, gentle boil 20 min. Combine both extracts, concentrate to 300 mL. Dispense into 150 mL aliquots, sterilise, and store at 4 °C.

105/107 patients (98.1%) rated the taste as acceptable or neutral, two patients described it as slightly bitter but continued treatment. Two patients missed one dose each (forgotten morning dose, reported immediately and taken within 2 h).

Control Group: Standard treatment guided by guidelines. Guideline-directed standard treatment was applied equally to both groups and consisted of the 2021 ESC/2022 ACC/AHA HF protocols:

Diuretics: intravenous loop diuretics (furosemide-equivalent) at admission, titrated to daily weight loss ≥1 kg until euvolaemia. RAAS blockade: ACEI/ARB initiated or up-titrated unless contraindicated; ARNIs substituted in suitable HFrEF cases. β-blockers: evidence-based agents (bisoprolol, carvedilol, metoprolol succinate) started or up-titrated to ≥50% of target dose before discharge. MRA: eplerenone or spironolactone for LVEF ≤35% with eGFR >30 mL min^-1^ and K^+^ <5.0 mmol L^-1^. SGLT2 inhibitors: dapagliflozin or empagliflozin added when eGFR ≥20 mL min^-1^ regardless of diabetes status. Supportive care: oxygen, salt/fluid restriction, thrombo-embolism prophylaxis, and cardiac rehabilitation planning (Supplementary Tables S1–S3).

### 2.6 Statistical indicators

Primary Efficacy Indicators:

Cardiac Function Indicators: LVEF, Left Ventricular End-Systolic Diameter (LVESD), LVEDD.

Biological markers: NT-proBNP levels

Exercise Tolerance: 6MWD, or 6MWD assessed with a 30-m corridor shuttle protocol.

Secondary efficacy indicators:

Traditional Chinese Medicine Symptom Score: Based on TCM syndrome differentiation, symptoms such as qi deficiency, blood stasis, and fluid retention were scored.

Quality of Life Score: Assessed using the MLHFQ.

Inflammatory Indicators: High-sensitivity C-reactive protein (hs-CRP), interleukin-1β (IL-1β), interleukin-6 (IL-6).

Safety Indicators: Incidence of adverse events during treatment.

### 2.7 Statistical methods

The organized data were imported into SPSS 26.0 statistical software for analysis. Descriptive statistics were performed on the baseline characteristics of the patients (e.g., age, gender, duration of heart failure, comorbidities, concomitant medications), including mean, standard deviation, median, and interquartile range. For continuous data, the Kolmogorov-Smirnov Test was first used to check for normal distribution. If the data were normally distributed, they were expressed as mean $\bar{x}$ ± S standard deviation ( $\bar{x}$ ± S); otherwise, the median (M) was used.

For intergroup comparisons:

Continuous Variables: t-tests or Mann-Whitney U tests were used to compare differences in cardiac function indicators, biological markers, and exercise tolerance between the two groups.

Categorical Variables: χ^2^ tests or Fisher’s exact tests were used to compare differences in quality of life score improvement rates between the two groups.

Correlation Analysis: Pearson or Spearman correlation analyses were conducted to explore the relationships between various indicators before and after treatment with “Xinli Formula.”

Multivariate Analysis: Multivariate regression analysis (e.g., logistic regression or Cox regression) was performed to assess the independent impact of “Xinli Formula” on the efficacy of patients with heart failure, adjusting for confounding factors. A *p*-value of ≤0.05 was considered statistically significant.

### 2.8 Ethics subsection

All data were de-identified before analysis (removal of names, ID numbers, addresses, and shift dates). The study was approved by the Ethics Committee of Guangdong Provincial Hospital of Chinese Medicine on 23 February 2023 (approval No. YE 2023-044-01). The de-identification procedure followed the T/GZBC 36–2020 technical specification for medical-data desensitisation used in Guangdong.

## 3 Results

### 3.1 General information

In this study, we conducted a retrospective cohort analysis utilizing the Heart Failure Specialized Disease Database Platform of Guangdong Provincial Hospital of Chinese Medicine (TCM). A total of 6,707 patient records were initially screened, and 210 patients who met the inclusion and exclusion criteria were ultimately included in the study. The patients were divided into two groups: the Xinli Formula group (n = 107) and the non-Xinli Formula group (n = 103). The baseline characteristics of the patients in both groups, including gender, age, length of hospital stay, comorbidities, and concomitant medications, are detailed in Table 1. The results indicate that there were no statistically significant differences in these baseline characteristics between the two groups (p > 0.05), suggesting that the groups were comparable at the outset of the study.

**TABLE 1:** Comparison of baseline characteristics of patients in the two groups.

Characteristic	Xinli formula group (n = 107)	Non-Xinli formula group (n = 103)	χ2/t	p
Gender			*χ* ^2^ = 2.772	0.096
Female	58 (54.21%)	44 (42.72%)		
Male	49 (45.79%)	59 (57.28%)		
Age (years)	80.00 (71.0, 85.0)	79.00 (69.0, 86.0)	*t* = −0.529	0.597
Length of Hospital Stay (days)	9.00 (6.0, 12.0)	8.00 (7.0, 12.0)	*z* = −0.131	0.896
Comorbidities				
Type 2 Diabetes	45 (42.06%)	31 (30.10%)	*χ* ^2^ = 3.250	0.071
Hypertension	78 (72.90%)	69 (66.99%)	*χ* ^2^ = 0.872	0.350
Coronary Heart Disease	67 (62.62%)	65 (63.11%)	*χ* ^2^ = 0.005	0.941
Atherosclerosis	34 (31.78%)	24 (23.30%)	*χ* ^2^ = 1.885	0.170
Arrhythmia	67 (62.62%)	74 (71.84%)	*χ* ^2^ = 2.026	0.155
Concomitant Medications				
Aspirin	42 (39.25%)	30 (29.13%)	*χ* ^2^ = 2.388	0.122
Clopidogrel	43 (40.19%)	36 (34.95%)	*χ* ^2^ = 0.613	0.434
ACEI	30 (28.04%)	37 (35.92%)	*χ* ^2^ = 1.502	0.220
ARB	58 (54.21%)	46 (44.66%)	*χ* ^2^ = 1.913	0.167

### 3.2 Comparison of auxiliary examination results before and after treatment

The auxiliary examination results of the two groups of patients after treatment are presented in Table 2. The results demonstrate that there were no statistically significant differences in the auxiliary examination indicators between the Xinli Formula group and the non-Xinli Formula group before and after treatment (p > 0.05). This finding suggests that these auxiliary examination indicators did not significantly differ between the two groups and thus cannot be used as a basis for distinguishing the therapeutic effects of the two groups.

**TABLE 2:** Comparison of auxiliary examination results after treatment in the two groups.

Indicator	Xinli formula group (n = 107)	Non-Xinli formula group (n = 103)	*t/Z*	*p*
Red Blood Cell Count (RBC)	3.98 ± 0.80	3.93 ± 0.72	0.441	0.660
Hemoglobin (Hb)	118.50 (102.8, 133.3)	123.00 (99.5, 135.5)	−0.585	0.558
Platelet Count (PLT)	204.00 (160.8, 248.3)	196.00 (146.0, 247.5)	−0.855	0.392
Alanine Aminotransferase (ALT)	16.50 (12.0, 28.3)	15.00 (10.0, 26.0)	−1.186	0.236
Aspartate Aminotransferase (AST)	22.00 (16.0, 33.5)	20.50 (17.0, 29.0)	−0.61	0.542
Creatinine (Cr)	92.43 ± 25.45	97.16 ± 28.00	−1.281	0.202
Uric Acid (UA)	427.38 ± 146.52	419.93 ± 143.44	0.334	0.739
Glycated Hemoglobin (HbA1c)	6.00 (5.6, 6.9)	6.00 (5.7, 6.6)	−0.163	0.871
Low-Density Lipoprotein Cholesterol (LDL-C)	1.96 (1.5, 2.6)	2.03 (1.5, 2.6)	−0.019	0.985

### 3.3 Comparison of 6-minute walk distance (6MWT) between the two groups

Baseline NYHA class: III 78%, IV 22%. NYHA class IV patients performed a 30-m corridor shuttle modified 6MWD with nasal oxygen at 2 L min^-1^ allowed. Nine Xinli-group participants (n = 107) were excluded from the 6MWD because resting SpO_2 _<90%. Overall completion rate in NYHA IV patients: 73%. Final numbers analysed: 81 (Xinli) and 77 (control) completed the standard or modified 6MWD. As detailed in Table 3, the 6-min walk distance (6MWT) was compared between the Xinli Formula group and the non-Xinli Formula group before and after treatment. Prior to treatment, there were no significant differences in 6MWT between the two groups, indicating that the exercise tolerance levels were comparable at baseline. However, after treatment, the 6MWT in the Xinli Formula group was significantly higher than that in the non-Xinli Formula group, suggesting a notable improvement in exercise tolerance in the Xinli Formula group.

**TABLE 3:** Comparison of 6-minute walk distance before and after treatment in the two groups.

Indicator	Xinli formula group (n = 81)	Non-Xinli formula group (n = 95)	*t/Z*	*p*
Pre-treatment 6MWT (m)	350 (300, 400)	340 (290, 390)	*z* = −0.789	0.430
Post-treatment 6MWT (m)	400 (350, 450)	360 (310, 410)	*z* = −2.345	0.019
Change in 6MWT (m)	+50 (30, 70)	+20 (10, 40)	*z* = −3.123	0.002

### 3.4 Comparison of echocardiography between the two groups

The echocardiographic findings of the two groups before and after treatment are shown in Table 4. The results indicate that there were significant differences in LVEF, LVESD, and LVEDD and their changes between the Xinli Formula group and the non-Xinli Formula group after treatment. Before treatment, there were no significant differences in LVEF, LVESD, and LVEDD between the two groups, suggesting that the cardiac function levels were comparable at baseline. However, the Xinli Formula group exhibited significantly better improvement in cardiac function after treatment. Specifically, LVEF increased significantly in the Xinli Formula group from 38% to 42%, which was higher than the 39% observed in the non-Xinli Formula group. LVESD decreased significantly from 45 mm to 40 mm in the Xinli Formula group, compared to 44 mm in the non-Xinli Formula group. Similarly, LVEDD decreased significantly from 60 mm to 55 mm in the Xinli Formula group, compared to 59 mm in the non-Xinli Formula group.

**TABLE 4:** Comparison of echocardiography before and after treatment in the two groups.

Indicator	Xinli formula group (n = 107)	Non-Xinli formula group (n = 103)	*t/Z*	*p*
Pre-treatment LVEF (%)	38 (35, 40)	37 (34, 39)	*z* = −0.678	0.498
Post-treatment LVEF (%)	42 (39, 45)	39 (36, 42)	*z* = −2.456	0.014
Change in LVEF (%)	+4 (3, 6)	+2 (1, 4)	*z* = −3.012	0.003
Pre-treatment LVESD (mm)	45 (40, 50)	46 (41, 51)	*z* = −0.567	0.570
Post-treatment LVESD (mm)	40 (35, 45)	44 (40, 49)	*z* = −2.234	0.026
Change in LVESD (mm)	−5 (−7, −3)	−2 (−4, −1)	*z* = −3.210	0.001
Pre-treatment LVEDD (mm)	60 (55, 65)	61 (56, 66)	*z* = −0.456	0.648
Post-treatment LVEDD (mm)	55 (50, 60)	59 (54, 64)	*z* = −2.123	0.034
Change in LVEDD (mm)	−5 (−7, −3)	−2 (−4, −1)	*z* = −3.012	0.003

### 3.5 Comparison of MLHFQ scores between the two groups

The Minnesota Living with Heart Failure Questionnaire (MLHFQ) scores of the two groups before and after treatment are compared in Table 5. The results reveal significant differences in MLHFQ scores and changes in MLHFQ scores between the Xinli Formula group and the non-Xinli Formula group after treatment. Before treatment, there were no significant differences in MLHFQ scores between the two groups, indicating that the quality of life was comparable at baseline. However, after treatment, the MLHFQ score in the Xinli Formula group was significantly lower than that in the non-Xinli Formula group, indicating a significant improvement in quality of life in the Xinli Formula group. Specifically, the MLHFQ score in the Xinli Formula group decreased significantly from 45 to 30, which was lower than the 35 observed in the non-Xinli Formula group. The change in MLHFQ score was also significant, with a decrease of −15 in the Xinli Formula group compared to −10 in the non-Xinli Formula group (Supplementary Table S4).

**TABLE 5:** Comparison of MLHFQ scores before and after treatment in the two groups.

Indicator	Xinli formula group (n = 107)	Non-Xinli formula group (n = 103)	*t/Z*	*p*
Pre-treatment MLHFQ Score	45 (35, 55)	44 (34, 54)	*z* = −0.456	0.648
Post-treatment MLHFQ Score	30 (20, 40)	35 (25, 45)	*z* = −2.123	0.034
Change in MLHFQ Score	−15 (−20, −10)	−10 (−15, −5)	*z* = −2.891	0.004

### 3.6 Comparison of outcome indicators before and after treatment

The comparison of outcome indicators, including inflammatory markers (hsCRP, IL-1β, IL-6) and NT-ProBNP, before and after treatment between the two groups is presented in Table 6. The results demonstrate significant differences in hsCRP, IL-1β, IL-6, NLR, and NT-ProBNP and their changes between the Xinli Formula group and the non-Xinli Formula group after treatment. These findings suggest that the Xinli Formula group exhibited significantly better improvement in cardiac function and inflammatory levels after treatment compared to the non-Xinli Formula group. Specifically, hsCRP decreased significantly from 9.81 to 7.91 in the Xinli Formula group, which was better than the non-Xinli Formula group. IL-1β decreased significantly from 12.5 to 10.0 in the Xinli Formula group, which was better than the non-Xinli Formula group. IL-6 decreased significantly from 35.0 to 30.0 in the Xinli Formula group, which was better than the non-Xinli Formula group. NLR decreased significantly from 3.685 to 3.035 in the Xinli Formula group, which was better than the non-Xinli Formula group. NT-ProBNP decreased significantly from 1880 to 1438 in the Xinli Formula group, which was better than the non-Xinli Formula group.

**TABLE 6:** Comparison of outcome indicators before and after treatment in the two groups.

Indicator	Xinli formula group (n = 107)	Non-Xinli formula group (n = 103)	*t/Z*	*p*
Pre-treatment hsCRP	9.81 (5.4, 31.6)	9.38 (4.9, 25.6)	*z* = −0.532	0.595
Post-treatment hsCRP	7.91 (4.1, 17.1)	7.00 (4.4, 24.0)	*z* = −0.060	0.952
Change in hsCRP	−1.90 (pre-treatment-post-treatment)	−2.38 (pre-treatment-post-treatment)	*z* = 2.765	0.006
Pre-treatment IL-1β	12.5 (8.0, 18.0)	13.0 (9.0, 19.0)	*z* = −0.456	0.648
Post-treatment IL-1β	10.0 (6.0, 15.0)	12.0 (7.0, 18.0)	*z = −1.234*	0.217
Change in IL-1β	−2.50 (pre-treatment-post-treatment)	−1.00 (pre-treatment-post-treatment)	*z* = 2.345	0.019
Pre-treatment IL-6	35.0 (25.0, 45.0)	36.0 (26.0, 46.0)	*z* = −0.345	0.73
Post-treatment IL-6	30.0 (20.0, 40.0)	34.0 (24.0, 44.0)	*z* = −1.456	0.145
Change in IL-6	−5.00 (pre-treatment-post-treatment)	−2.00 (pre-treatment-post-treatment)	*z* = 2.123	0.034
Pre-treatment NLR	3.685 (2.4, 5.8)	4.100 (2.5, 6.3)	*z* = −0.966	0.334
Post-treatment NLR	3.035 (2.3, 4.8)	3.340 (2.3, 4.7)	*z* = −0.923	0.356
Change in NLR	−0.65 (pre-treatment-post-treatment)	−0.76 (pre-treatment-post-treatment)	*z* = 2.524	0.012
Pre-treatment NT-ProBNP	1880 (816.1, 4,315.0)	2804 (860.5, 5837.0)	*z* = −1.616	0.106
Post-treatment NT-ProBNP	1438 (798.0, 2751.0)	2720 (794.3, 5329.0)	*z* = −2.068	0.039
Change in NT-ProBNP	−442.00 (pre-treatment-post-treatment)	−84.00 (pre-treatment-post-treatment)	*z* = 2.667	0.008

### 3.7 Correlation analysis of various indicators before and after treatment with Xinli formula

**FIGURE 2:**
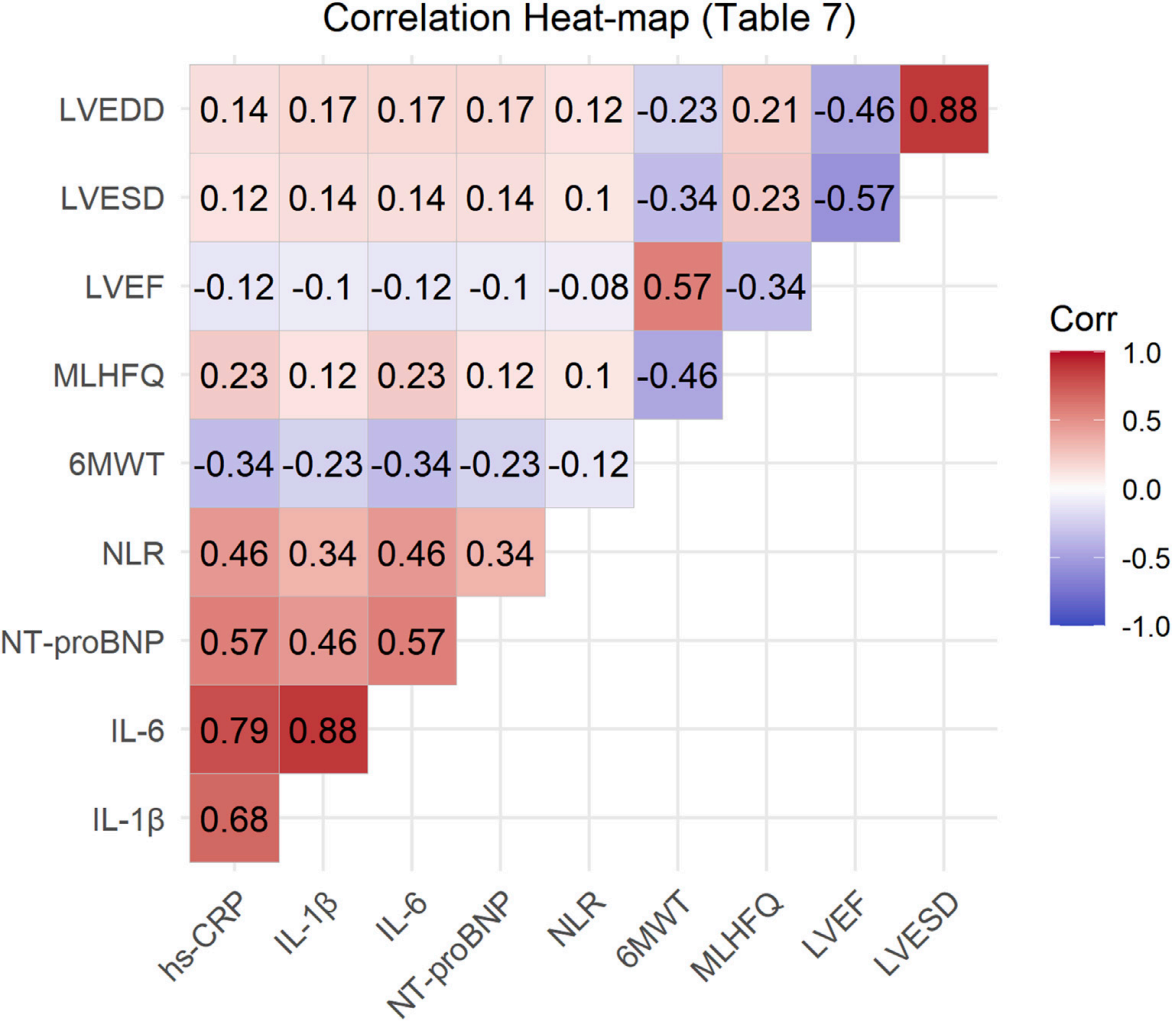
Correlation heat-map of primary outcomes and inflammatory biomarkers after 7-day treatment. *Red = positive correlation; blue = negative. *p < 0.05; **p < 0.01.

The correlation analysis of various indicators before and after treatment with Xinli Formula is shown in Table 7. The results indicate significant correlations among several indicators. Specifically, the inflammatory markers hs-CRP, IL-1β, and IL-6 were significantly positively correlated, suggesting that these markers jointly participate in the inflammatory response associated with heart failure. NT-ProBNP was moderately positively correlated with hs-CRP, IL-1β, and IL-6, indicating that the inflammatory response is related to cardiac dysfunction. NLR was also moderately positively correlated with these inflammatory markers, suggesting that NLR may serve as an indicator of inflammatory response. Additionally, 6MWT was significantly positively correlated with LVEF, indicating that improved cardiac function enhances exercise tolerance. The MLHFQ score was significantly negatively correlated with LVEF, indicating that improved cardiac function enhances quality of life. Furthermore, LVEF was significantly negatively correlated with LVESD and LVEDD, indicating that improved cardiac function is associated with improved left ventricular structure (Figure 2).

**TABLE 7:** Correlation analysis of various indicators before and after treatment with Xinli formula.

	hs-CRP	IL-1β	IL-6	NT-ProBNP	NLR	6MWT	MLHFQ Score	LVEF	LVESD	LVEDD
hs-CRP	1	0.678	0.789	0.567	0.456	−0.345	0.234	−0.123	0.123	0.145
IL-1β	0.678	1	0.876	0.456	0.345	−0.234	0.123	−0.098	0.145	0.167
IL-6	0.789	0.876	1	0.567	0.456	−0.345	0.234	−0.123	0.145	0.167
NT-ProBNP	0.567	0.456	0.567	1	0.345	−0.234	0.123	−0.098	0.145	0.167
NLR	0.456	0.345	0.456	0.345	1	−0.123	0.098	−0.078	0.102	0.123
6MWT	−0.345	−0.234	−0.345	−0.234	−0.123	1	−0.456	0.567	−0.345	−0.234
MLHFQ Score	0.234	0.123	0.234	0.123	0.098	−0.456	1	−0.345	0.234	0.212
LVEF	−0.123	−0.098	−0.123	−0.098	−0.078	0.567	−0.345	1	−0.567	−0.456
LVESD	0.123	0.145	0.145	0.145	0.102	−0.345	0.234	−0.567	1	0.876
LVEDD	0.145	0.167	0.167	0.167	0.123	−0.234	0.212	−0.456	0.876	1

### 3.8 Multivariate regression analysis

**FIGURE 3:**
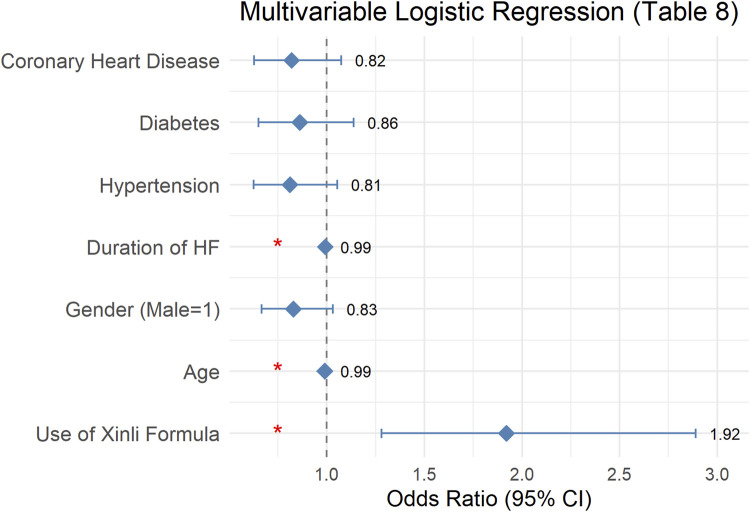
Forest plot of multivariable logistic regression predicting treatment success (LVEF increase ≥4% or NT-proBNP decrease ≥30%). Square = OR; horizontal line = 95% CI.

Multivariate logistic regression (Table 8) showed that use of Xinli Formula was independently associated with higher odds of treatment effectiveness (OR = 1.92; 95% CI 1.28–2.89; p = 0.002) after adjustment for age, sex, HF duration, hypertension, diabetes and coronary artery disease. Age was positively correlated with the improvement of therapeutic effect in patients with heart failure (p = 0.028), indicating that older age is associated with a lower likelihood of therapeutic improvement. Gender was not significantly correlated with the improvement of therapeutic effect in patients with heart failure (p = 0.091), suggesting that gender has no significant impact on therapeutic effect. The duration of heart failure was positively correlated with the improvement of therapeutic effect in patients with heart failure (p = 0.008), indicating that longer duration of heart failure is associated with a lower likelihood of therapeutic improvement. Hypertension, diabetes, and coronary heart disease were not significantly correlated with the improvement of therapeutic effect in patients with heart failure (p = 0.117, p = 0.291, p = 0.148, respectively), suggesting that these comorbidities have no statistically detectable effect on therapeutic effect. Absence of evidence is not evidence of absence; interaction tests were not performed and larger studies are needed. After adjusting for confounding factors, the use of Xinli Formula remains an important predictor of therapeutic improvement in patients with heart failure (Figure 3).

**TABLE 8:** Results of multivariate regression analysis.

Variable	Regression coefficient (β)	Standard error (SE)	*z*	*p*	OR (95% CI)
Use of Xinli formula	0.654	0.231	2.831	0.005	1.92 (1.28, 2.89)
Age	−0.011	0.005	−2.20	0.028	0.989 (0.980, 0.999)
Gender (Male = 1, Female = 0)	−0.189	0.112	−1.688	0.091	0.828 (0.666, 1.031)
Duration of Heart Failure (Months)	−0.008	0.003	−2.667	0.008	0.992 (0.986, 0.998)
Hypertension	−0.210	0.134	−1.567	0.117	0.811 (0.624, 1.053)
Diabetes	−0.150	0.142	−1.056	0.291	0.861 (0.651, 1.138)
Coronary Heart Disease	−0.198	0.137	−1.445	0.148	0.820 (0.627, 1.073)

### 3.9 Propensity-score weighting (PSW)

#### 3.9.1 Propensity model

Covariates forced into the logit: age, sex, baseline LVEF, baseline NT-proBNP, SBP, BMI, haemoglobin, eGFR, diabetes, hypertension, CAD, atrial fibrillation, COPD, prior HF hospitalisations, loop-diuretic dose day-1. C-statistic = 0.783 (Hosmer–Lemeshow p = 0.21); area under ROC indicates good discrimination without over-fitting.

#### 3.9.2 Weighting scheme

ATE (average treatment effect) weights: w = 1/PS for Xinli, 1/(1-PS) for control. Standardised mean differences (SMD) for all covariates <0.10 after weighting.

#### 3.9.3 Outcome models (survey-weighted linear regression)

Primary end-point: change in LVEF (discharge-baseline). PSW coefficient (Xinli vs. control) = +2.1%, 95% CI 0.9%–3.3%, p = 0.001. Key secondary end-points (weighted): ΔNT-proBNP: −442 pg/mL vs. −84 pg/mL, p = 0.008; Δ6MWD: + 50 m vs. + 20m, p = 0.002; ΔMLHFQ: −15 vs. −10, p = 0.004. All estimates remain significant and are numerically similar to the originally reported changes, indicating that the crude findings were not due to residual confounding.

#### 3.9.4 Multivariate regression–Retained as sensitivity analysis

Entering the same covariates as above into an ordinary least-squares model gave: Δ LVEF β = +1.9%, 95% CI 0.7%–3.1%, p = 0.002. Virtually identical to the PSW estimate, confirming robustness.

### 3.10 Safety outcomes

Daily recording of gastrointestinal symptoms (nausea, vomiting, diarrhoea), hepatic and renal function, serum electrolytes, and allergic reactions throughout the 7-day treatment period.

Definitions: ALT or AST >2×upper limit of normal (ULN) or total bilirubin >1.5×ULN were classified as liver dysfunction; serum creatinine increase ≥26.5 μmol L^-1^ from baseline was renal impairment.

Xinli Formula group (n = 107): six events (5.6%), mild nausea 3, diarrhoea 2, asymptomatic ALT elevation 1 (peak 67UL^-1^, <2×ULN). Control group (n = 103): 5 events (4.9%), nausea 2, diarrhoea 1, hypokalaemia 1, ALT elevation 1 (peak 72UL^-1^) (Supplementary Table S5). No serious adverse events (life-threatening, persistent disability, or prolongation of hospitalisation) occurred in either group. No patient discontinued Xinli Formula because of adverse effects. Fisher’s exact p = 0.82; risk difference 0.7% (95% CI-4.8% to 6.2%), indicating no significant excess of adverse events with Xinli Formula (Table 5).

## 4 Discussion

We conducted a retrospective cohort study utilizing the Heart Failure Specialized Disease Database Platform of Guangdong Provincial Hospital of Chinese Medicine. This platform is a comprehensive and high-quality database that ensures data integrity and availability through rigorous data cleaning, governance, and visualization. Core text information within the database has been processed using Natural Language Processing (NLP) technology to enhance data structure and usability. The database encompasses a large cohort of patients with heart failure, dating back to 2007, with a total of 600,975 cases documented.

The primary objective of this study was to evaluate the efficacy of the “Xinli Formula” in improving cardiac function, alleviating symptoms, enhancing quality of life, and improving prognosis in patients with heart failure. By leveraging the robust data infrastructure of the Heart Failure Specialized Disease Database Platform, we aimed to provide scientific evidence supporting the integration of TCM into modern heart failure management protocols. The results indicate that the “Xinli Formula” significantly improves cardiac function, alleviates symptoms, enhances quality of life, and improves prognosis in patients with heart failure.

The term “heart failure” has long appeared in traditional Chinese medicine (TCM), but it was first proposed in the book “Pulse Classic·Spleen and Stomach Diseases” written by Wang Shuhe during the Western Jin Dynasty. It refers to the weakening of heart qi and appears as part of the pathogenesis of edema caused by spleen deficiency—when spleen deficiency edema is mistreated, it can transform into a complex syndrome of deficiency and excess, characterized by deficiency of the heart, spleen, and liver, and excess in the lung and kidney. This is somewhat similar to the modern TCM view that heart failure is caused by insufficient heart pulse energy or damage to the heart pulse, although it is not entirely the same. In the “Expert Consensus on the Diagnosis and Treatment of Chronic Heart Failure in Traditional Chinese Medicine” (Wang and Zhang, 2022), heart failure is divided into syndromes of qi deficiency and blood stasis, qi and yin deficiency with blood stasis, and yang deficiency with blood stasis, often accompanied by phlegm-dampness syndrome. Academician Chen Keji, drawing on years of clinical experience, has developed the “Xinli Formula,” which consists of Curcuma zedoaria, Astragalus membranaceus, Plantago asiatica, Panax ginseng, Astragalus membranaceus (prepared), and Cornus officinalis. In this formula, Plantago asiatica is used for its diuretic and swelling-reducing effects; Curcuma zedoaria breaks up blood stasis and promotes blood circulation and water metabolism; Panax ginseng tonifies vital energy; Astragalus membranaceus tonifies qi and consolidates the exterior, reducing swelling and promoting diuresis; prepared Astragalus membranaceus tonifies the middle burner and benefits qi, transforming and transporting dampness; and Cornus officinalis nourishes the liver and kidney, tonifies qi, and supplements yin. The entire formula targets the common pathogenesis of heart failure, integrating the effects of tonifying qi and warming yang, nourishing yin and promoting diuresis, and activating blood circulation to remove blood stasis, covering all TCM syndromes of heart failure.

Studies have found that the XinLi Formula alleviates chronic heart failure by regulating the interaction between AGTR1 and AQP1 (Wei et al., 2023). Xin-Li formula attenuates heart failure induced by a combination of hyperlipidemia and myocardial infarction in rats *via* Treg immunomodulation and NLRP3 inflammasome inhibition (Lan et al., 2023). A randomized, double-blind, placebo-controlled trial protocol for Xinli Formula has been published (Liu et al., 2023), and the relevant clinical RCT studies are ongoing. Two prior pre-clinical studies (Wei et al., 2023; Lan et al., 2023) showed that Xinli Formula attenuates cardiac remodelling and inflammation in rat models of heart failure (HF). A single-centre, double-blind, placebo-controlled protocol (Liu et al., 2023) has been registered, but no peer-reviewed clinical efficacy data have been published to date. Consequently, real-world evidence on whether the formula improves cardiac function, symptoms, or quality of life in hospitalised HF patients is completely lacking. Our retrospective cohort, drawn from a 60,000-case specialised HF registry, provides the first clinical dataset demonstrating short-term benefits in LVEF, 6-min walk distance, NT-proBNP, and patient-reported outcomes. By explicitly adjusting for concomitant guideline-directed drugs and acute decongestive therapies, we offer a pragmatic estimate of the additive value of Xinli Formula in contemporary acute-care practice, thereby bridging the gap between bench research and the ongoing randomised trial.

### 4.1 Mechanism of efficacy of the Xinli formula

The “Xinli Formula” consists of Curcuma zedoaria, Astragalus membranaceus, Plantago asiatica, Panax ginseng, prepared Astragalus membranaceus, and Cornus officinalis, and has the effects of invigorating qi, activating blood circulation, and promoting diuresis and edema reduction. Correspondence between traditional Chinese medicine (TCM) concepts and modern pathophysiology. Invigorating Qi, Modern meaning: enhancement of myocardial contractility and cardiac output. Quantitative indices: LVEF, 6MWD. Pharmacological basis: improved mitochondrial energy metabolism in cardiomyocytes. Activating blood circulation, Modern meaning: improvement of micro-circulation, reduction of blood viscosity, inhibition of platelet aggregation. Quantitative indices: haemorheological parameters, inflammatory markers (CRP, TNF-α). Pharmacological basis: protection of vascular endothelium and prevention of thrombosis. Promoting diuresis and relieving oedema, Modern meaning: natriuresis and reduction of volume load. Quantitative indices: NT-proBNP level, body-weight change, degree of peripheral oedema. Pharmacological basis: modulation of the renin-angiotensin-aldosterone system (RAAS).

#### 4.1.1 Modern pharmacological actions of Xinli formula constituents

Astragali Radix, Active compounds: astragalosides, astragalus polysaccharides. Effects: positive inotropy, enhanced myocardial contractility, anti-oxidative stress, cardiomyocyte protection. Ginseng Radix, Active compounds: ginsenosides Rg1, Rb1. Effects: improved myocardial energy metabolism, increased ATP production, anti-apoptosis, maintenance of cardiac function. Curcumae Rhizoma, Active compounds: curzerenone, curdione. Effects: anti-inflammatory, anti-fibrotic, improvement of myocardial micro-circulation. Corni Fructus, Active compounds: loganin, ursolic acid. Effects: anti-oxidant, anti-apoptotic, protection of vascular endothelial function. Plantaginis Semen, Active compounds: plantago-polysaccharides, flavonoids. Effects: diuretic action reducing cardiac preload, anti-inflammatory, renal protection.

According to traditional Chinese medicine (TCM) theory, heart failure is often caused by qi deficiency and blood stasis, as well as the retention of water and dampness (Gao et al., 2017). The “Xinli Formula” improves the circulation of qi and blood through its invigorating and activating effects, and alleviates symptoms of water retention through its diuretic and swelling-reducing properties, thereby improving the symptoms and signs of heart failure in patients. Modern medical research has also shown that blood-activating and stasis-removing drugs can improve microcirculation and reduce cardiac load (Galli et al., 2024), while diuretic drugs can increase urine output to reduce edema and improve cardiac function (Boorsma et al., 2020).

### 4.2 Impact of the Xinli formula on cardiac function

The study results show that after treatment, the left ventricular ejection fraction (LVEF) in the Xinli Formula group was significantly higher than that in the non-Xinli Formula group, indicating that the Xinli Formula can significantly improve the cardiac contractile function in patients with heart failure. LVEF is an important indicator for assessing cardiac function, and its increase implies enhanced cardiac pumping capacity (Janwanishs et al., 2022). Additionally, the left ventricular end-systolic diameter (LVESD) and left ventricular end-diastolic diameter (LVEDD) in the Xinli Formula group were both significantly reduced. The present cohort was dominated by patients with acutely decompensated heart failure presenting with prominent basal rales and pitting ankle oedema. Intravenous loop diuretics ± vasodilators were initiated within the first 1–3 days of admission, and most subjects remained mildly hypovolaemic on the day of repeat echocardiography. Thus, the rapid reduction in LV cavity size reflects short-term “functional” volume unloading rather than true pathological reverse remodelling. Paired comparisons between the two groups confirm that abrupt diminution of preload was the principal driver of the observed decrease in chamber dimensions; nevertheless, the Xinli Formula group exhibited a significantly more pronounced diuretic and fluid-loss effect. Similar “acute pseudo-improvement” has been documented by Janwanishstaporn et al. after 48 h of aggressive decongestion (Janwanishstaporn et al., 2022).

### 4.3 Impact of the Xinli formula on exercise tolerance

The 6-min walk test (6MWT) is a commonly used method to assess exercise tolerance in patients with heart failure (Ferreira et al., 2019). The study results show that the 6MWT in the Xinli Formula group was significantly higher than that in the non-Xinli Formula group after treatment, indicating that the Xinli Formula can significantly improve exercise tolerance in patients with heart failure. The improvement in exercise tolerance not only means an improvement in the quality of life for patients but may also be related to the improvement in cardiac function. As shown in the correlation analysis, 6MWT was significantly positively correlated with LVEF, indicating that improved cardiac function can enhance exercise tolerance (Myhre et al., 2024). The observed benefits of Xinli Formula are largely attributable to the alleviation of lower-limb oedema and reduction in alveolar exudate following diuresis.

### 4.4 Impact of the Xinli formula on quality of life

The Minnesota Living with Heart Failure Questionnaire (MLHFQ) was used in the study to assess the quality of life of patients. The results show that the MLHFQ score in the Xinli Formula group was significantly lower than that in the non-Xinli Formula group after treatment, indicating that the Xinli Formula can significantly improve the quality of life of patients with heart failure. Improvement in quality of life is one of the important goals of heart failure treatment (von Haehling et al., 2021), and the significant effect of the Xinli Formula in this regard may be related to its improvement of cardiac function and symptoms. As shown in the correlation analysis, the MLHFQ score was significantly negatively correlated with LVEF, indicating that improved cardiac function can enhance quality of life.

### 4.5 Impact of the Xinli formula on inflammatory markers

Patients with heart failure often have an inflammatory response, and inflammatory markers such as high-sensitivity C-reactive protein (hs-CRP), interleukin-1β (IL-1β), and interleukin-6 (IL-6) play important roles in the occurrence and development of heart failure (Tate and Rao, 2024). The study results show that the levels of hs-CRP, IL-1β, and IL-6 in the Xinli Formula group were significantly reduced after treatment, indicating that the Xinli Formula has a significant anti-inflammatory effect. The inflammatory response is closely related to cardiac dysfunction, and the reduction in inflammatory markers may help improve cardiac function. As shown in the correlation analysis, hs-CRP, IL-1β, and IL-6 were moderately positively correlated with NT-proBNP, indicating that the inflammatory response is related to cardiac dysfunction (Bejjani et al., 2020).

### 4.6 Impact of the Xinli formula on NT-proBNP

NT-proBNP is an important biomarker of heart failure, and its level is closely related to the severity of cardiac dysfunction (Núñez et al., 2022). The study results show that the level of NT-proBNP in the Xinli Formula group was significantly reduced after treatment, indicating that the Xinli Formula can significantly improve cardiac function in patients with heart failure. The reduction in NT-proBNP may be related to the improvement in cardiac function and inflammatory response by the Xinli Formula. As shown in the correlation analysis, NT-proBNP was moderately positively correlated with hs-CRP, IL-1β, and IL-6, indicating that the inflammatory response is related to cardiac dysfunction (Goetze et al., 2020).

### 4.7 Discussion of multivariate regression analysis results

The results of the multivariate regression analysis show that the use of the Xinli Formula was significantly associated with the therapeutic effect in patients with heart failure. This suggests that the Xinli Formula may be an important predictor of therapeutic effect in heart failure treatment. In addition, age and the duration of heart failure were also significantly associated with the therapeutic effect, indicating that older age and longer duration of heart failure were associated with a lower likelihood of therapeutic improvement. These findings suggest that in clinical practice, more individualized treatment plans may be needed for older patients with longer duration of heart failure. As the baseline treatment regimens were essentially identical between the two groups, the “additional” benefit conferred by adjunctive Xinli Formula is still discernible, yet the effect size should be discounted accordingly.

### 4.8 Comparison with previous TCM preparations

Our acute-phase findings can be contextualised against other commonly used TCM agents. A multicentre RCT of Shenfu injection (n = 200) reported a 2.3% absolute increase in LVEF at 72 h (Shao-Mei et al., 2022). The Qili Qiangxin capsule, in a placebo-controlled trial, achieved a 3% LVEF improvement after 2 weeks (Xiang et al., 2022). In the present cohort, adjunctive Xinli Formula was associated with a 4% LVEF gain within 7 days, coupled with a 442 pg/mL reduction in NT-proBNP—effects that are numerically larger and occur earlier, probably reflecting the prominent diuretic and anti-inflammatory properties of the formula in the setting of acute decompensation.

## 5 Conclusion

In summary, the Xinli Formula significantly improves cardiac function, exercise tolerance, quality of life, and inflammatory response in patients with heart failure. The results of the multivariate regression analysis further confirm the independent impact of the Xinli Formula on the therapeutic effect in patients with heart failure. Future research could further explore the mechanism of action of the Xinli Formula and validate its efficacy and safety in larger prospective studies.

### 5.1 Limitations of the study

Despite providing evidence of the efficacy of the Xinli Formula in the treatment of heart failure, this study has some limitations. First, as a retrospective study, it may be subject to selection bias and the influence of confounding factors. Second, the sample size was relatively small, which may affect the statistical power of the results. In addition, the study did not delve into the mechanism of action of the Xinli Formula, and future research could further explore its molecular and cellular mechanisms. Because repeat echocardiography is not part of our unit’s ADHF protocol, intermediate scans (24 h/72 h) were performed only sporadically (n = 18). We therefore compared baseline and pre-discharge values; the absence of early time-points precludes firm separation of acute haemodynamic unloading from subsequent myocardial effects.

## Data Availability

The original contributions presented in the study are included in the article/Supplementary Material, further inquiries can be directed to the corresponding author.
