# Supplementary material for: Xinli formula’s efficacy in heart failure: analysis via Guangdong TCM hospital database
Source: Front Pharmacol. 2025 Nov 17;16:1661625. doi: 10.3389/fphar.2025.1661625 (PMC12665773; doi:10.3389/fphar.2025.1661625)
Supplement: Supplementary file 1 [file Table1.docx]

### 1 Intravenous loop diuretics (first 72 h)

| Variable | Xinli group (n = 107) | Non-Xinli group (n = 103) | P value |
| --- | --- | --- | --- |
| First i.v. furosemide equivalent median [IQR], mg | 80 [60–120] | 80 [60–120] | 0.91 |
| ≤ 40 mg | 18 (16.8 %) | 20 (19.4 %) | 0.62 |
| > 120 mg | 21 (19.6 %) | 19 (18.4 %) | 0.84 |
| Cumulative dose day-1, mg | 200 [160–280] | 190 [150–260] | 0.33 |
| Cumulative dose day-2, mg | 340 [280–440] | 320 [260–420] | 0.29 |
| Torasemide switch | 12 (11.2 %) | 10 (9.7 %) | 0.73 |
| Bumetanide switch | 5 (4.7 %) | 4 (3.9 %) | 0.76 |
| Thiazide sequential | 14 (13.1 %) | 13 (12.6 %) | 0.92 |
| Acetazolamide add-on | 6 (5.6 %) | 5 (4.9 %) | 0.82 |

2-h spot urine Na ≥ 50 mmol/L: 71 % vs 69 %, P = 0.70

6-h urine output ≥ 100 mL/h: 66 % vs 66 %, P = 0.99

24-h weight loss, kg (mean ± SD): 1.3 ± 0.7 vs 1.2 ± 0.8, P = 0.41

Interpretation: Both arms received equivalent loop-diuretic exposure and showed similar natriuretic and weight-loss responses during the first 72 h.

### 2 Inotropes / vasopressors (0–72 h)

| Drug | Dose range | Xinli (n, %) | Control (n, %) | P |
| --- | --- | --- | --- | --- |
| Dobutamine | 2–8 μg kg⁻¹ min⁻¹ | 15 (14.0 %) | 13 (12.6 %) | 0.76 |
| Dopamine* | ≤ 5 μg kg⁻¹ min⁻¹ | 9 (8.4 %) | 11 (10.7 %) | 0.56 |
| Milrinone | 0.25–0.5 μg kg⁻¹ min⁻¹ | 8 (7.5 %) | 7 (6.8 %) | 0.83 |
| Levosimendan | 12 μg kg⁻¹ bolus → 0.1 μg kg⁻¹ min⁻¹ | 5 (4.7 %) | 4 (3.9 %) | 0.76 |
| Norepinephrine | 0.05–0.2 μg kg⁻¹ min⁻¹ | 3 (2.8 %) | 4 (3.9 %) | 0.64 |

*Low-dose dopamine for renal vasodilation, not pressor.

Indication: SBP < 90 mmHg or cardiac index < 2.2 L min⁻¹ m⁻² with pulmonary capillary wedge pressure > 18 mmHg. Median duration 24 h [18–36 h]; no inter-group difference.

### 3 Vasodilators (0–72 h)

| Drug | Dose range | Xinli (n, %) | Control (n, %) | P |
| --- | --- | --- | --- | --- |
| Nitroglycerin i.v. | 5–100 μg min⁻¹ | 33 (30.8 %) | 31 (30.1 %) | 0.92 |
| Sodium nitroprusside | 0.2–2 μg kg⁻¹ min⁻¹ | 11 (10.3 %) | 9 (8.7 %) | 0.7 |

4 MLHFQ domain-specific changes (n = 204 after imputation)

| Domain | Xinli group Δ [95 % CI] | Control group Δ [95 % CI] | Pinteraction |
| --- | --- | --- | --- |
| Physical | −6.5 [−8.2, −4.8] | −3.9 [−5.6, −2.2] | 0.011 |
| Emotional | −3.2 [−4.6, −1.8] | −2.9 [−4.3, −1.5] | 0.78 |
| Respiratory | −7.2 [−9.0, −5.4] | −4.1 [−5.9, −2.3] | 0.007 |

5 Hepatic safety – CTCAE v5.0 grading

| Parameter | CTCAE grade* | Xinli (n = 107) | Control (n = 103) | P† |
| --- | --- | --- | --- | --- |
| ALT | G1 | 1 (0.9 %) | 1 (1.0 %) | 1 |
| AST | G1 | 0 (0 %) | 1 (1.0 %) | 0.49 |
| Total bilirubin | G0 | 107 (100 %) | 103 (100 %) | — |
| Creatinine↑ | G1 | 2 (1.9 %) | 3 (2.9 %) | 0.68 |
| Hypokalaemia | G1 | 3 (2.8 %) | 4 (3.9 %) | 0.72 |
| Nausea | G1 | 3 (2.8 %) | 2 (1.9 %) | 1 |
| Diarrhoea | G1 | 2 (1.9 %) | 1 (1.0 %) | 1 |

*G0 = normal; G1 = mild (e.g., ALT > ULN–3×ULN, Cr increase ≥ 26.5 µmol/L and reaching Grade 1 threshold).

†Fisher’s exact test; no Grade ≥ 2 events occurred in either arm.
